# Supplementary material for: Multi-environment analysis enhances genomic prediction accuracy of agronomic traits in sesame
Source: Front Genet. 2023 Mar 13;14:1108416. doi: 10.3389/fgene.2023.1108416 (PMC10040590; doi:10.3389/fgene.2023.1108416)
Supplement: Supplementary file 1 [file Table1.pdf]

Multi-environment analysis enhances genomic prediction accuracy of agronomic traits in sesame

Idan Sabag[1], Ye Bi[2], Zvi Peleg[1*], and Gota Morota[2*]

[1]The Robert H. Smith Institute of Plant Sciences and Genetics in Agriculture, The Hebrew University of Jerusalem, Rehovot 7610001, Israel
[2]School of Animal Sciences, Virginia Polytechnic Institute and State University, Blacksburg, VA, USA

[*] Corresponding author
E-mail: morota@vt.edu and zvi.peleg@mail.huji.ac.il

Supplementary Materials

Tables

Table S1: List of the genotypes and their country of origin.

Genotype	Continent	Country	Genotype	Continent	Country
S-1	Asia	Israel	S-314	North America	Nicaragua
S-2	Asia	Turkey	S-315	Europe	Greece
S-3	Africa	Ethiopia	S-316	Europe	Bulgaria
S-4	South America	Bolivia	S-317	Europe	Bulgaria
S-8	Asia	Israel	S-318	Asia	Nepal
S-9	Africa	Ethiopia	S-319	Asia	Nepal
S-10	Africa	Ethiopia	S-320	Asia	Chile
S-11	Africa	Ethiopia	S-321	Asia	China
S-25	Africa	B. Fasso	S-322	Asia	Sri Lanka
S-27	Africa	Somalia	S-323	Asia	Iran
S-31	Africa	Ethiopia	S-324	Asia	Sri Lanka
S-33	North America	USA	S-325	Africa	Egypt
S-34	Africa	Egypt	S-327	Asia	Jordan
S-36	Asia	Pakistan	S-328	Asia	Israel
S-37	Asia	Thailand	S-329	Asia	Saudi Arabia
S-38	Africa	Egypt	S-331	Asia	Yemen
S-39	Asia	Afghanistan	S-332	Africa	Sudan
S-40	North America	Mexico	S-333	Asia	Soviet
S-41	North America	USA	S-334	Asia	Soviet
S-44	Asia	Soviet	S-335	Africa	Nigeria
S-45	Africa	Egypt	S-336	Asia	Nepal
S-46	North America	USA	S-337	Asia	Iran
S-47	North America	USA	S-338	North America	USA
S-48	Africa	Kenya	S-339	Asia	Israel
S-49	Asia	Turkey	S-340	Asia	India
S-50	Africa	Egypt	S-341	South America	Venezuela
S-51	Africa	Sudan	S-342	Africa	Congo
S-52	North America	USA	S-408	Unknown	Unknown
S-54	Africa	Nigeria	S-416	Asia	Israel
S-55	Asia	Japan	S-417	Asia	Israel
S-56	Asia	Turkey	S-423	South America	Peru
S-58	Asia	Israel	S-428	North America	Mexico
S-59	Asia	Iran	S-431	Africa	Nigeria
S-60	South America	Argentina	S-432	Africa	Nigeria
S-61	Africa	Egypt	S-433	Africa	Nigeria
S-62	Africa	Sudan	S-434	Asia	Pakistan
S-63	Asia	Jordan	S-435	Asia	Pakistan
S-64	Europe	Greece	S-443	Africa	Egypt

Genotype	Continent	Country	Genotype	Continent	Country
S-65	Africa	Egypt	S-445	Africa	Egypt
S-67	Europe	Cyprus	S-447	Africa	Egypt
S-68	Asia	Syria	S-448	Asia	China
S-70	Africa	Egypt	S-450	Asia	China
S-71	Africa	Congo	S-451	Asia	China
S-73	Asia	India	S-452	Africa	Mozambique
S-74	Asia	Iraq	S-454	Africa	Mozambique
S-75	Asia	Pakistan	S-456	Asia	Korea
S-78	Africa	Nigeria	S-459	Asia	Israel
S-79	Africa	Kenya	S-460	Asia	Israel
S-80	North America	USA	S-462	Asia	Israel
S-81	North America	Mexico	S-463	Asia	Israel
S-82	North America	USA	S-464	Asia	Israel
S-83	North America	USA	S-466	Asia	Israel
S-85	North America	USA	S-467	Asia	Sri Lanka
S-86	North America	USA	S-472	Europe	Greece
S-87	Europe	Greece	S-473	Europe	Greece
S-88	Asia	Soviet	S-474	Europe	Greece
S-89	Africa	Kenya	S-475	Europe	Greece
S-90	Africa	Libya	S-476	Asia	Japan
S-91	Africa	Egypt	S-478	Asia	Japan
S-92	Asia	Afghanistan	S-479	Asia	Thailand
S-93	South America	Peru	S-481	Asia	Thailand
S-95	Asia	Iraq	S-482	Asia	Thailand
S-96	Unknown	Unknown	S-487	South America	Venezuela
S-97	Europe	Greece	S-488	South America	Venezuela
S-98	North America	USA	S-490	Europe	Bulgaria
S-100	Asia	China	S-491	Europe	Bulgaria
S-102	Asia	Soviet	S-493	Asia	Indonesia
S-104	North America	Mexico	S-494	Asia	Indonesia
S-106	North America	Virgin island	S-496	Asia	Nepal
S-109	Asia	India	S-497	Asia	Nepal
S-112	Asia	Iran	S-500	Asia	Yemen
S-113	South America	Venezuela	S-501	Asia	Yemen
S-114	North America	Mexico	S-503	Africa	Sudan
S-115	Asia	India	S-504	Asia	Iran
S-117	Asia	Jordan	S-508	Asia	Afghanistan
S-118	Unknown	Unknown	S-509	Unknown	Unknown
S-121	Unknown	Unknown	S-510	Unknown	Unknown
S-123	Asia	Israel	S-515	Unknown	Unknown
S-126	South America	Venezuela	S-520	Unknown	Unknown
S-127	Unknown	unknown	S-522	Unknown	Unknown
S-129	North America	USA	S-525	Unknown	Unknown
S-134	Asia	Israel	S-528	Unknown	Unknown

Genotype	Continent	Country	Genotype	Continent	Country
S-135	Asia	Israel	S-529	Unknown	Unknown
S-136	North America	USA	S-534	Unknown	Unknown
S-137	Asia	Israel	S-550	Asia	India
S-138	Asia	Israel	S-554	Africa	Mozambique
S-278	North America	Mexico	S-570	Africa	Mozambique
S-297	South America	Venezuela	S-575	Africa	Nigeria
S-305	Asia	Taiwan	S-576	Africa	Nigeria
S-311	Africa	Sudan	S-577	Africa	Ethiopia
S-312	Asia	Turkey	S-581	South America	Paraguay

Table S2: Mean, standard deviation (SD), coefficient of variation (CV), and heritability estimates (H^2) of the nine agronomic sesame traits.

**Trait**	**2018**			**2020**			
	Mean	SD	CV(%)	Mean	SD	CV(%)	H^2
Flowering date	51.05	8.93	17.5	50.54	7.97	15.8	0.97
Height to the first capsule	71.59	28.66	40	92.45	38.74	41.9	0.96
Plant height	136.13	18.4	13.5	184.93	27.86	15.1	0.89
Reproductive zone	64.74	19.21	29.6	92.92	22.36	24.1	0.91
Reproductive index	0.48	0.15	32.7	0.51	0.14	28.6	0.97
Number of branches per plant	5.17	2.43	47.1	3.94	2.08	52.9	0.88
Seed-yield per plant	17.09	7.41	43.4	18.63	6.56	35.2	0.74
Seeds number per plant	3545	1558	44	6351.14	2204	34.7	0.66
Thousand-seed weight	3.12	0.5	16.3	2.94	0.4	13.6	0.88

Table S3: Single-environment genomic prediction accuracies of the best linear unbiased prediction (GBLUP), BayesB, BayesC, and reproducing kernel Hilbert spaces (RKHS) regression models obtained from repeated random sub-sampling cross-validation replicated 50 times. Mean is the mean value of the prediction accuracies, SD is their standard deviation and P-value is the significance obtained by the analysis of variance among the regressions models.

**Trait**	**Model**	**2018**			**2020**		
		Mean	SD	P-value	Mean	SD	P-value
Flowering date	GBLUP	0.74	0.04	0.33	0.71	0.06	0.89
	BayesB	0.74	0.04		0.71	0.06	
	BayesC	0.74	0.04		0.71	0.06	
	RKHS	0.72	0.05		0.7	0.07	
Height to the first capsule	GBLUP	0.77	0.04	0.35	0.79	0.04	0.76
	BayesB	0.77	0.04		0.79	0.04	
	BayesC	0.77	0.04		0.79	0.04	
	RKHS	0.75	0.06		0.78	0.04	
Plant height	GBLUP	0.68	0.05	0.83	0.67	0.06	0.14
	BayesB	0.68	0.05		0.67	0.06	
	BayesC	0.68	0.05		0.67	0.06	
	RKHS	0.69	0.04		0.65	0.06	
Reproductive zone	GBLUP	0.56	0.07	0.46	0.54	0.06	0.58
	BayesB	0.56	0.07		0.54	0.07	
	BayesC	0.56	0.07		0.54	0.07	
	RKHS	0.54	0.09		0.52	0.07	
Reproductive index	GBLUP	0.71	0.05	0.31	0.75	0.05	0.69
	BayesB	0.71	0.05		0.75	0.05	
	BayesC	0.72	0.05		0.75	0.05	
	RKHS	0.7	0.07		0.74	0.05	
No. of branches per plant	GBLUP	0.69	0.05	0.87	0.63	0.07	0.5
	BayesB	0.69	0.05		0.63	0.07	
	BayesC	0.69	0.05		0.63	0.07	
	RKHS	0.68	0.05		0.61	0.08	
Seed-yield per plant	GBLUP	0.56	0.06	0.99	0.4	0.1	0.98
	BayesB	0.57	0.06		0.4	0.1	
	BayesC	0.56	0.06		0.4	0.1	
	RKHS	0.68	0.05		0.39	0.09	
Seeds number per plant	GBLUP	0.56	0.06	0.82	0.41	0.1	0.95
	BayesB	0.57	0.06		0.4	0.09	
	BayesC	0.56	0.06		0.41	0.1	
	RKHS	0.57	0.07		0.4	0.08	
Thousand-seed weight	GBLUP	0.55	0.09	0.97	0.51	0.08	0.75
	BayesB	0.55	0.09		0.51	0.08	
	BayesC	0.55	0.09		0.51	0.08	
	RKHS	0.55	0.08		0.5	0.09	

Table S4: Multi-environment genomic prediction accuracies of the marker-by-environment interaction model obtained from repeated random sub-sampling cross-validation replicated 50 times. Mean is the mean value of the prediction accuracies, and SD is their standard deviation. P-value was obtained from the corrected resampled t-test comparing to the single-environment genomic best linear unbiased prediction model.

**Trait**	**Year**	**CV1**			**CV2**		
		Mean	SD	P-value	Mean	SD	P-value
Flowering date	2018	0.74	0.05	0.29	0.92	0.02	$< .0001$
	2020	0.73	0.06	0.36	0.91	0.03	.0002
Height to the first capsule	2018	0.77	0.05	0.36	0.91	0.02	$< .0001$
	2020	0.78	0.05	0.45	0.91	0.02	.0004
Plant height	2018	0.67	0.05	0.28	0.82	0.03	$< .0001$
	2020	0.66	0.06	0.42	0.8	0.04	.0008
Reproductive zone	2018	0.55	0.08	0.24	0.8	0.04	$< .0001$
	2020	0.52	0.08	0.40	0.77	0.05	.0001
Reproductive index	2018	0.71	0.06	0.29	0.88	0.03	$< .0001$
	2020	0.74	0.05	0.44	0.89	0.03	.0004
No. of branches per plant	2018	0.7	0.06	0.43	0.84	0.03	$< .0001$
	2020	0.63	0.07	0.49	0.78	0.05	.01
Seed-yield per plant	2018	0.58	0.07	0.18	0.68	0.06	.001
	2020	0.43	0.09	0.36	0.58	0.08	.03
Seeds number per plant	2018	0.51	0.08	0.31	0.6	0.07	0.003
	2020	0.41	0.1	0.46	0.52	0.08	0.1
Thousand-seed weight	2018	0.54	0.1	0.35	0.82	0.04	$< .0001$
	2020	0.48	0.09	0.37	0.81	0.03	$< .0001$

Figures

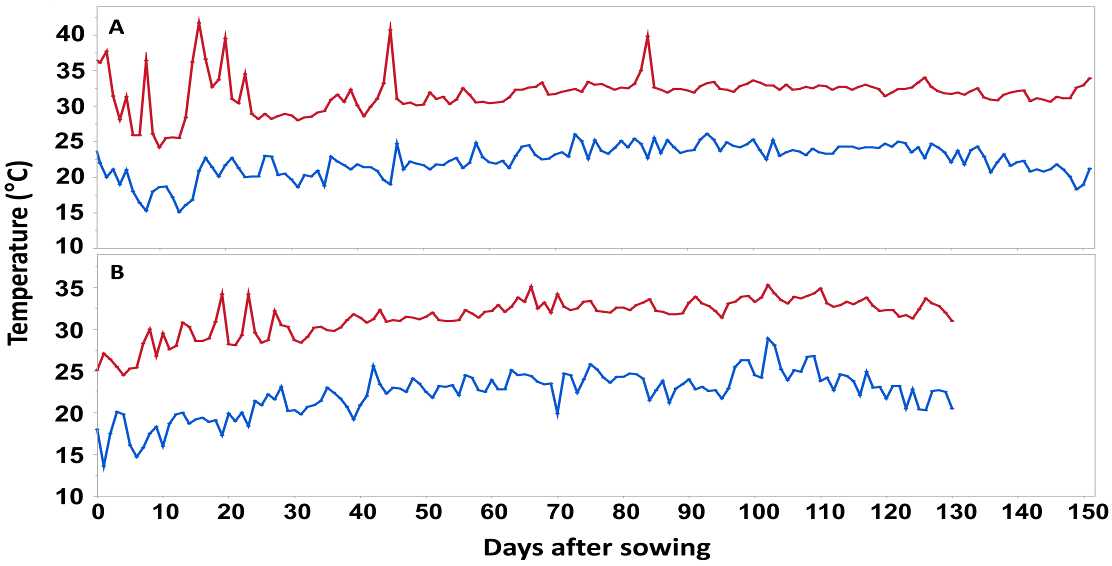

Figure S1: Maximum (red) and minimum (blue) temperatures during the 2018 (A) and 2020 (B) growing seasons. The number of growing days was 150 and 130, respectively.
